# Structural deficits and cognitive impairment in tuberculous meningitis

**DOI:** 10.1186/s12879-015-1011-z

**Published:** 2015-07-22

**Authors:** Hsiu-Ling Chen, Cheng-Hsien Lu, Ching-Di Chang, Pei-Chin Chen, Meng-Hsiang Chen, Nai-Wen Hsu, Kun-Hsien Chou, Wei-Ming Lin, Ching-Po Lin, Wei-Che Lin

**Affiliations:** Department of Diagnostic Radiology, Kaohsiung Chang Gung Memorial Hospital, and Chang Gung University College of Medicine, 123 Ta-Pei Road, Niao-Sung, Kaohsiung 83305 Taiwan; Department of Biomedical Imaging and Radiological Sciences, National Yang-Ming University, Taipei, Taiwan; Department of Neurology, Kaohsiung Chang Gung Memorial Hospital and Chang Gung University College of Medicine, Kaohsiung, Taiwan; Department of Radiology, Yuan’s General Hospital, Kaohsiung, Taiwan; Institute of Neuroscience, National Yang-Ming University, Taipei, Taiwan; Department of Diagnostic Radiology, Chang Gung Memorial Hospital at Chiayi, Chang Gung University College of Medicine, Chiayi, Taiwan

**Keywords:** Tuberculous meningitis, Cognitive deficits, Magnetic resonance imaging, Voxel-based morphometry

## Abstract

**Background:**

Chronic neuropsychological sequelae may occur in patients with tuberculous meningitis (TBM). The impact of structural abnormalities on the clinical performance of patients with TBM is unknown. This study applied the Diffeomorphic Anatomical Registration Through Exponentiated Lie Algebra (DARTEL) voxel-based morphometry (VBM) to determine if gray matter deficits in TBM are associated with acute presentations and chronic cognitive impairment.

**Methods:**

Seventeen patients with TBM who discontinued their anti-TB therapy for more than six months, and 17 age-, sex-, and education-matched healthy subjects were enrolled. Differences in gray matter volume (GMV) between patients and healthy controls were investigated using DARTEL-VBM to determine structural abnormalities. Disease severity during the acute stage was scored by clinical profiles and conventional imaging findings. Correlations among chronic structural deficits, cognitive impairment, and initial disease severity were assessed.

**Results:**

The patients with TBM had worse neuropsychological subtest performances than the healthy controls. Compared to the controls, the patients showed smaller GMVs in the right thalamus, right caudate nucleus, right superior and middle temporal gyrus, right precuneus, and left putamen (*p* < 0.001). The smaller GMVs in the right thalamus, right superior temporal gyrus, right precuneus, left putamen, and right caudate nucleus (*p* < 0.05) were further associated with worse cognitive function. More severe initial disease also correlated with smaller GMVs in the right caudate nucleus (*p* < 0.05).

**Conclusion:**

Multiple domain cognitive impairment may persist in patients with chronic TBM even after appropriate treatment. Worse initial disease severity may contribute to the vulnerability of brain tissue to damage, with subsequent neuropsychological consequences.

## Background

Tuberculous meningitis (TBM) remains a serious health threat in developing countries. Chronic neuropsychological sequelae may occur even after appropriate treatment, often in the form of cognitive impairment, motor deficits, and optic atrophy [1, 2]. Many prognostic factors for TBM have been reported, including age, disease stage, level of consciousness, the presence of extra-central nervous system (CNS) TB, the isolation of Mycobacterium tuberculosis (M. tuberculosis) from cerebrospinal fluid (CSF), CSF biochemical studies, hydrocephalus, and infarction [3–5]. Nonetheless, the mechanisms that lead to brain structural and functional abnormalities and that mediate cognitive and behavioural outcomes in TBM remain unclear.

Clinical neuropsychiatric performances have been shown to be highly correlated with brain structural integrity in many diseases and conditions [6, 7]. However, there are no previous studies that have reported on the association between brain structural deficits and cognitive impairment in TBM. At the same time, it is interesting to know the induced factors related to brain atrophy. Several indices in the acute stage, including clinical presentations and imaging findings, are considered to be related to disease prognosis [4]. Voxel-based morphometry (VBM) has previously been used to evaluate brain volume in cases of bacterial meningitis [8, 9], as it can help evaluate anatomic brain abnormalities in varying subgroups. This study applied the Diffeomorphic Anatomical Registration Through Exponentiated Lie Algebra (DARTEL) VBM [10] to determine gray matter deficits in TBM during the chronic stage under the hypothesis that disease severity in the acute stage can predict long-term structural abnormality and cognitive function impairment.

## Methods

### Subjects

From January 2009 to December 2010, 24 HIV-negative TBM patients who had discontinued their anti-TB therapy and had been discharged from the hospital for more than six months were seen at the Neurology Out-patient Clinic of Chang Gung Memorial Hospital-Kaohsiung Medical Centre. Among those patients, 17 were enrolled in this study and received both neuroimaging and neuropsychological follow-up examinations. The hospital’s Institutional Review Committee on Human Research approved the study, and all the patients provided written informed consent.

The criteria used to diagnose TBM were the same as those used in a previously published study [11]. TBM was defined as clinical presentations of chronic meningitis with (1) positive CSF mycobacterial culture and/or positive polymerase chain reaction (isolation of M. tuberculosis); or (2) isolation of M. tuberculosis outside the central nervous system (CNS) with typical CSF features [12]. The exclusion criteria for this study included age <16 or >80 years, evidence of alcoholism, a history of neurologic or psychiatric illness potentially affecting the CNS, and severe recent life events which could affect the results of the neuropsychiatric or neuroimaging surveys.

The patients’ medical records were reviewed to obtain the following information: demographic characteristics, underlying diseases, clinical features, laboratory data, bacteriology, imaging studies, surgical interventions or drainage, and clinical outcomes. Most of the patients had CSF samples taken on admission, and these samples were tested for total cell count and for glucose, protein, and lactate levels, in addition to being subjected to a polymerase chain reaction for TB (TB-PCR). The patients were also tested with mycobacterial smears and cultures.

Cranial computed tomography (CT) scans and/or magnetic resonance imaging (MRI) studies were done on admission, and repeat CT and/or MRI was conducted if there was clinical deterioration before discharge. Hydrocephalus was diagnosed by the presence of a dilated temporal horn of the lateral ventricle without obvious brain atrophy and/or an Evan’s ratio > 0.3 on CT or MRI during admission [13]. Evan’s ratio is defined as the ratio between the ventricular width of the bilateral frontal horn and the maximum internal bi-parietal diameter [14]. Patients with hydrocephalus and with evidence of increasing intra-cerebral pressure or clinical deterioration underwent a ventriculo-peritoneal shunting procedure [15].

All of the patients underwent complete medical and neurologic examinations, and neuropsychological testing. Experienced neurologists integrated the clinical manifestations and neuropsychological findings. For comparison, 17 sex-, age-, and education-matched healthy subjects without a medical history of neurologic disease, psychiatric disease, or head injury were recruited through a hospital advertisement and served as the control group.

### Evaluation of disease severity in acute stage

Significant variables with predictive value for TBM diagnosis were analyzed, and two different diagnostic scores were evaluated (Table 1), including clinical profile and conventional MRI variables. The clinical profile variables which were significant for TBM presumption were used. The cut-off was the suggestive value determined by previous studies [1, 4, 16], and a score of 1 or 2 was considered predictive for TBM.

**Table 1:** Scoring of disease severity indices of patients with TBM during acute presentation

	Characteristics	Score	Value	No.		Characteristics	Score	Value	No.
Clinical profile variables					Conventional MRI variables				
(1)	CSF/Blood glucose ratio	2	<0.6	2	(1)	Evan’s ratio score	2	>0.3	10
		1	≧0.6	15			1	<0.3	7
(2)	CSF WBC (/mm^3^)	2	≧300	3	(2)	Lepto-meningeal enhancement	2	Yes	9
		1	<300	14			1	No	8
(3)	CSF Lactate (mg/dl)	2	≧35	8	(3)	Basilar enhancement	2	Yes	2
		1	<35	9			1	No	15
(4)	CSF Total protein (g/L)	2	≧150	8	(4)	Parenchymal lesion	2	Yes	4
		1	<150	9			1	No	13
(5)	GCS on admission	2	<13	2	(5)	Infarction	2	Yes	5
		1	≧13	15			1	No	12
(6)	Age (years)	2	≧50	11					
		1	<50	6					

In terms of the conventional MRI variables [1], hydrocephalus (Evan’s ratio score > 0.3) [17], lepto-meningeal enhancement, basilar enhancement, parenchymal lesion [18], and infarction were used. A score of 2 indicated a positive finding, and a score of 1 indicated a negative finding.

The total disease severity scores consisted of the summation of the clinical profile and conventional MRI scores. Thus, higher scores indicated more severe disease during the acute presentation.

### Neuropsychological (NP) tests

Both the patients and healthy controls were administered subtests of the Wechsler Adult Intelligence Scale (WAIS). The WAIS, a family of tests of cognitive domains contributing to intelligence created by David Wechsler (Wechsler, 1955, 1981, 1997), is used to assess a wide range of cognitive abilities and impairments. In this study, we used the full scale intelligence quotient measure from the Chinese version of the WAIS-III [19, 20], which is based on the combined perceptual organization (POI), verbal comprehension (VCI), working memory (WMI), and processing speed index (PSI) scores [21]. All of the participants completed the subtests, including the block design, picture completion, matrix reasoning, vocabulary, similarities, information, digit span, arithmetic, and letter-number sequence subtests, that together comprise the POI, VCI, and WMI scores individually.

### MRI data acquisition

Magnetic resonance scanning was performed on a 3 T MRI system (Excite; GE Medical System) equipped with an eight-channel head coil. High-resolution T1-weighted imaging was acquired parallel to the anterior commissure-posterior commissure line (AC-PC line) using three-dimensional fluid-attenuated inversion-recovery fast spoiled gradient echo sequence. The parameters were TR = 9.492 ms, TE = 3.888 ms, TI = 450 ms, flip angle = 20°, field of view (FOV) = 24×24 cm, matrix size = 512×512, 110 continuous slices with the slice thickness = 1.3 mm, and in-plane spatial resolution of 0.47×0.47 mm.

### Imaging data processing

Individual T1-weighted data was processed using the VBM8 toolbox [22] (http://dbm.neuro.uni-jena.de/vbm/) under Statistical Parametic Mapping (SPM8, Wellcome Institute of Neurology, University College London, UK) implemented in Matlab 7.3 (MathWorks, Natick, MA, USA). A T1 VBM method based on DARTEL was used for preprocessing [10, 23] and subsequent analyses as follows: first, each native T1-weighted image was segmented into gray matter (GM), white matter (WM), and CSF. Second, the individual native space tissue segments were initially affine registered to the tissue probability maps in the Montreal Neurological Institute (MNI) space. Third, these tissue segments were iteratively registered to the group template, which was generated from all the images included in the study. Then, to modulate the GM and WM tissue for group volume comparisons, the nonlinear deformation parameters derived from the previous normalization procedure were used. The final resolution of each modulated tissue segment was 1.5 × 1.5 × 1.5 mm^3^. Finally, all segmented, normalized, and modulated GM images were smoothed with an 8-mm Gaussian kernel before voxel-wise group comparisons. The GM, WM, and CSF volumes were calculated by counting the voxels represented in the native space and estimated in cm^3^. The total intracranial volume (TIV) was determined from the sum of the aforementioned three volumes.

### Statistical analysis

#### Analysis of demographic data

Statistical analysis was performed by using SPSS 12 (SPSS Inc, Chicago, IL). All data were given as the mean ± standard deviation (SD). Demographic and clinical characteristics of the TBM and control groups were compared by independent *t*-test and analysis of variance (ANOVA) (for age and education). Analysis of covariance (ANCOVA) was performed to compare TIV, gray matter volume (GMV), and white matter volume (WMV), with age and sex as covariates. Statistical differences in neuropsychological data (WAIS) between groups were estimated by ANCOVA, with age, sex, and education as covariates. Statistical significance was set at *p* < 0.05.

#### Analysis of regional GMV differences between groups

To investigate regional GMV differences between the groups, a voxel-wise general linear model was used and an ANCOVA was performed with age, sex, and TIV as covariates. To avoid possible partial volume effects between different tissue types, voxels with a GM probability lower than 0.2 were excluded. In order to correct for the non-isotropic smoothness of the data, non-stationary correction (part of the VBM toolbox) was used to investigate group differences [24]. The voxel-wise level threshold was set to an uncorrected p < 0.001 with a cluster size >50 contiguous voxels and a nonstationary cluster extent threshold of p < 0.05 corrected for multiple comparisons to obtain precise findings.

To transform MNI coordinates into Talairach coordinates in order to minimize transformation discrepancies between MNI and the Talairach space, the GingerALE toolbox (The BrainMap Development Team; available online at http://brainmap.org/ale/index.html) was used. The Talairach and Tournoux atlas [25] was used to identify the anatomic structures of the coordinates representing significant clusters.

#### Correlation analysis

Partial correlation analysis adjusted for age, sex, education, and TIV was performed to assess the correlations among the total disease severity scores during acute presentation, the neuropsychological testing scores, and locations that exhibited smaller volumes in the TBM group compared to the control group. Regional GMV was extracted from the peak coordinate and correlated with the total disease severity scores and neuropsychological testing scores separately. The statistical significance threshold was set at *p* < 0.05.

## Results

### Clinical characteristics between groups

There were 34 subjects in this study, including 17 patients with TBM and 17 healthy controls. Their baseline characteristics, neuroimaging information, and cognitive function data are summarized in Table 2. Of the 17 patients, 13 (76.47 %) were male. The patients had a mean age of 50.76 ± 18.97 years (range: 17–78 years) and a median follow-up duration of 41 months (range: 9–161 months). There were no significant differences in age, sex, and education between the patients and controls.

**Table 2:** Demographic characteristics of patients with TBM and healthy controls

	TBM patients	Healthy controls	F	*p* value
Number of cases	17	17		
Sex (*n* = male/female)	13 / 4	13 / 4		
Age (years)	50.76 ± 18.97	50.41 ± 17.20	0.153	0.698
Education (years)	9.18 ± 5.11	13.18 ± 4.91	0.291	0.594
Total intracranial volume (TIV) (cm^3^)	1491.16 ± 120.01	1572.95 ± 115.23	0.099	0.755
Gray matter (GM)	676.41 ± 55.64	717.43 ± 50.31	0.579	0.452
White matter (WM)	495.33 ± 46.12	528.74 ± 35.04	0.676	0.417
Cerebrospinal fluid (CSF)	319.42 ± 34.31	326.79 ± 40.71	1.415	0.243
WAIS				
Picture Completion	7.18 ± 3.49	11.71 ± 4.37	4.126	0.051
Vocabulary	8.82 ± 3.38	12.12 ± 2.93	2.786	0.106
Digit Symbol	7.00 ± 3.03	12.12 ± 3.10	14.130	0.001*
Similarities	7.59 ± 3.32	11.53 ± 3.04	4.917	0.035*
Block Design	8.29 ± 2.95	11.82 ± 3.19	4.988	0.033*
Arithmetic	8.06 ± 2.84	11.12 ± 2.40	4.139	0.051*
Matrix Reasoning	8.12 ± 3.92	11.71 ± 2.93	4.334	0.046*
Digit Span	8.50 ± 3.98	11.35 ± 3.06	1.683	0.205
Information	8.18 ± 2.46	11.41 ± 3.54	2.122	0.156
Picture Arrangement	7.62 ± 3.46	11.47 ± 4.20	3.359	0.077*
Comprehension	8.65 ± 3.43	12.35 ± 3.30	2.997	0.094
Letter-Number Sequencing	6.40 ± 4.22	10.43 ± 3.76	4.501	0.044
Verbal comprehension (VCI)	90.24 ± 14.71	108.82 ± 16.22	4.258	0.048*
Perceptual organization (POI)	87.53 ± 18.45	111.29 ± 19.49	6.009	0.020*
Working memory (WMI)	85.25 ± 18.48	105.06 ± 16.78	4.829	0.036*

Age and education data were determined via independent *t*-test

TIV data given were determined by ANCOVA after controlling for age, sex, and education; GM, WM, and CSF data were determined by ANCOVA after controlling for age, sex, education, and TIV

NP test data were determined by ANCOVA after controlling for age, sex, education

*p < 0.05

### Disease severity scoring during acute stage in TBM

At the time of admission during the acute disease, all 17 TBM patients received complete laboratory tests and a conventional MRI study to assess the disease. Their clinical features and laboratory data (Table 1), including low CSF/serum glucose ratios (<0.6, *n* = 2), high CSF white cell counts (≥300/mm^3^, *n* = 3), high CSF lactate concentrations (≥35 mg/dl, *n* = 8), high CSF protein concentrations (≥150 g/L, *n* = 8), decreased Glasgow coma scores (GCS) on admission (<13, *n* = 2), and old age (≥50 years, *n* = 11), indicated poor prognosis.

Conventional neuroimaging results during the acute disease were also recorded, and these results indicated a variety of issues, including hydrocephalus (Evan’s ratio >0.3, *n* = 10), lepto-meningeal enhancement (*n* = 9), basilar enhancement (*n* = 2), parenchymal lesion (*n* = 4), and cerebral infarct (*n*= 5).

### Cognitive profiles in chronic stage between groups

In the chronic stage, all the patients and healthy subjects underwent neuropsychological testing and an MRI study, which included T1-weighted volumetric imaging. The patients with TBM performed significantly poorer on the digit symbol, similarities, block design, matrix reasoning, and letter-number sequencing subtests of the WAIS compared to the controls (*p* < 0.05). The patients with TBM had worse VCI, POI, and WMI results (Table 2).

### Chronic regional gray matter volume (GMV) differences between groups

Conventional MRI findings in the chronic stage included hydrocephalus (*n* = 3), persistent basilar enhancement (*n* = 2), old infarcts (*n* = 5), an old haemorrhage (*n* = 1), and a peri-ventricular signal change (*n* = 1).

The DARTEL volumetric analysis revealed significant differences in GMVs, with specific locations and extent of regions (Table 3).

**Table 3:** Regions with significantly smaller GMVs in patients with TBM compared to healthy controls

Gray matter volume	Anatomical regions	x	y	z	Brodmann area	Cluster size	T-value
Control > TBM	R Thalamus	17	-24	6	-	246	4.87
	R Superior Temporal Gyrus	48	11	-30	38	187	4.55
	R Precuneus	29	-75	48	7	163	4.22
	R Middle Temporal Gyrus	62	-51	2	22	334	4.08
	L Putamen	-17	15	0	-	118	4.03
	R Caudate nucleus	15	17	-8	-	58	3.82
	R Middle temporal Gyrus	56	-33	-12	21	71	3.67
TB > Control	None						

Voxel-based morphometry of TBM patients compared to healthy controls at an uncorrected *p* value <0.001 and cluster size >50 contiguous voxels, and a nonstationary correction for multiple comparison

Each T-value was determined by dividing the estimated regression coefficient by its standard error. "-" indicated that the Brodmann area was not available

*Abbreviations*: *R* Right; *L* Left

**Fig. 1:**
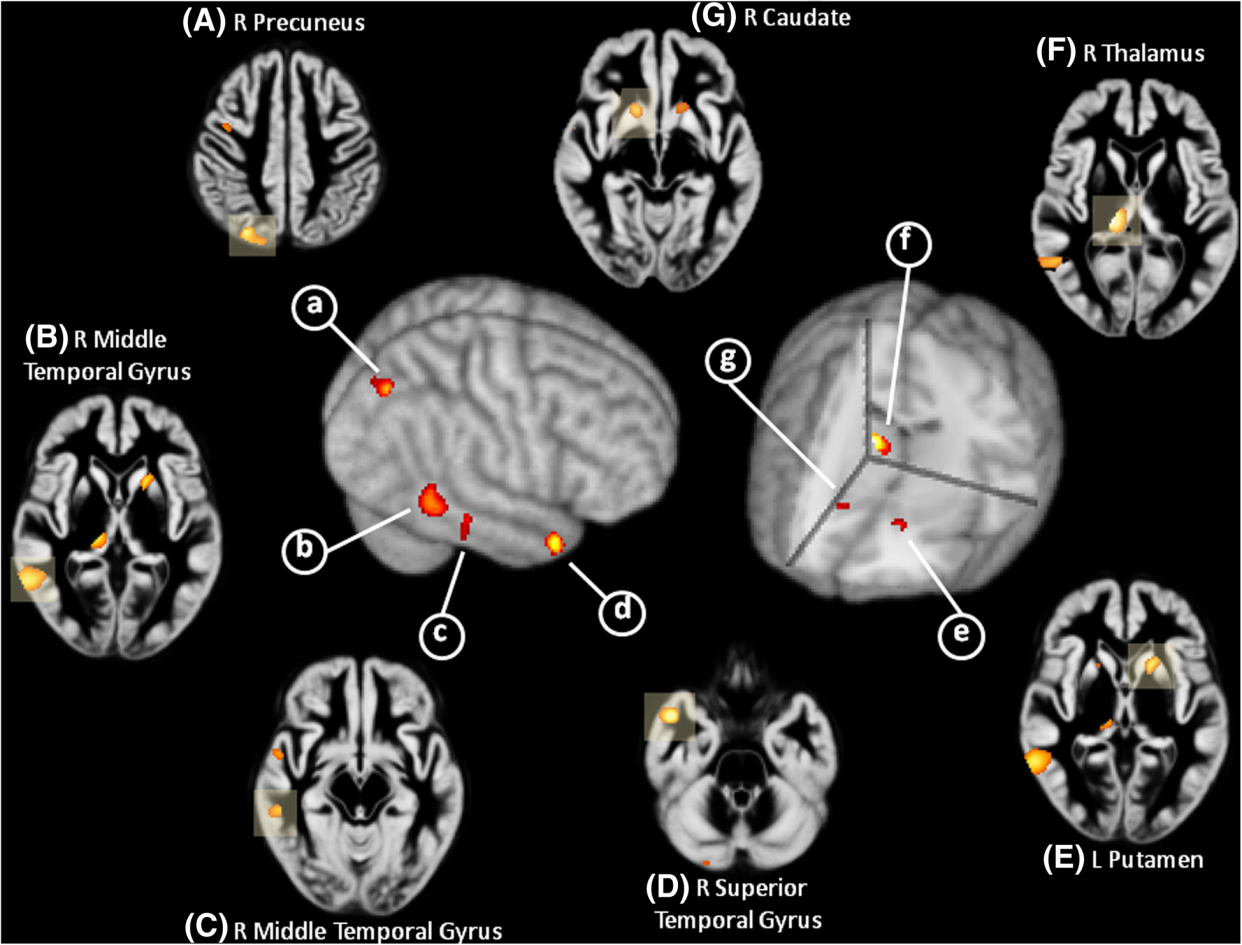
Results of the VBM comparisons between patients with TBM and healthy controls. On 3D renderings of the brain and representative axial slices through the customized template, patients with TBM had smaller gray matter volumes (GMVs), with highlighted significant areas in the right thalamus, right superior and middle temporal gyrus, right precuneus, and right caudate nucleus

Patients with TBM showed significantly smaller GMVs in the right thalamus, right superior temporal gyrus (BA 38), right precuneus (BA 7), right middle temporal gyrus (BA 22), left putamen, right caudate nucleus, and right middle temporal gyrus (BA 21) (*p* < 0.001, uncorrected) compared to the controls (Fig. 1).

### Relationship between cognitive function, disease severity, and gray matter volumes

**Fig. 2:**
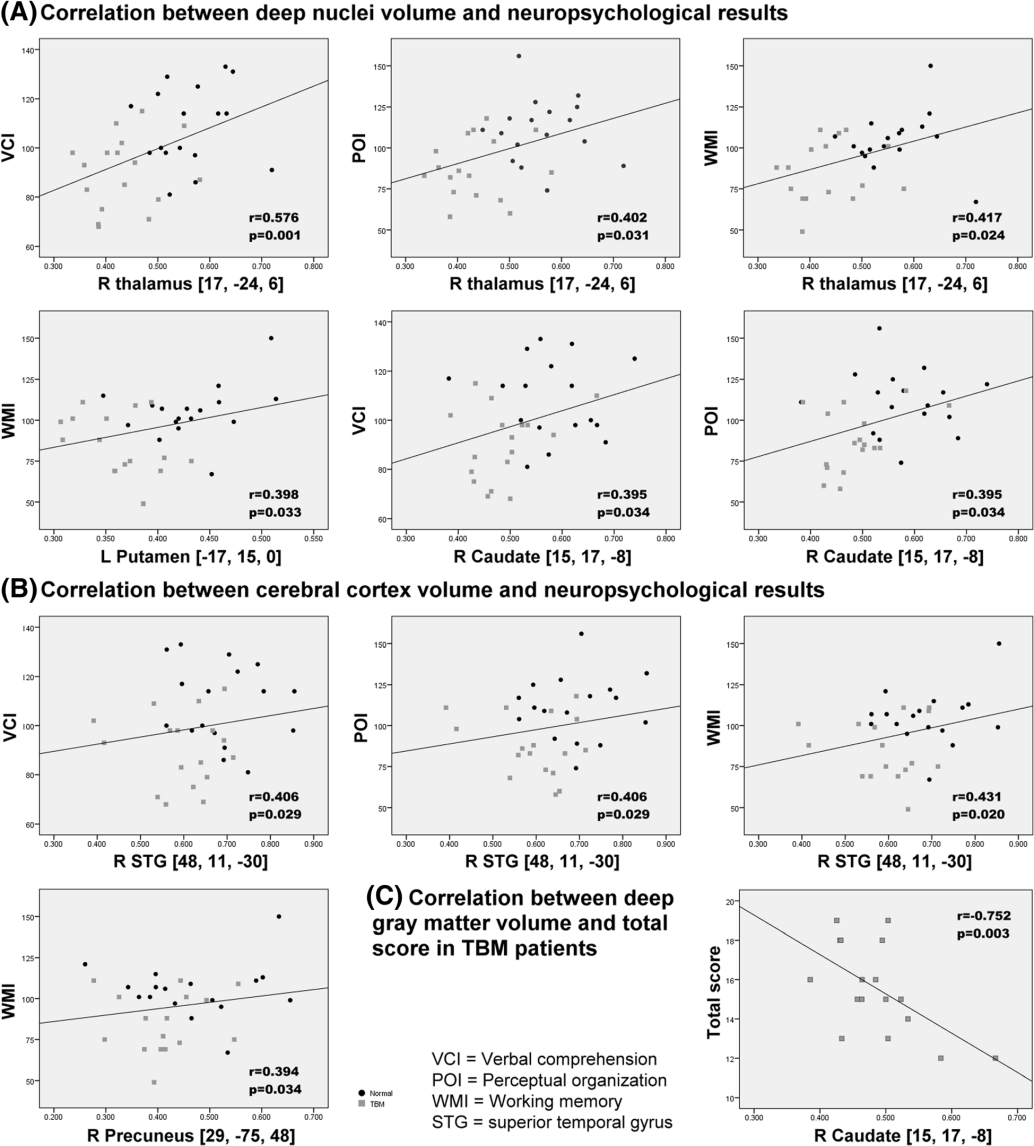
Significant partial correlations (*p* < 0.05) were plotted for (**a**) deep nuclei and (**b**) cerebral cortex cluster volumes in relation to the NP results (including verbal comprehension, perceptual organization, and working memory). The strongest correlation was between the right thalamus cluster volume and the verbal comprehension index. **c** In TBM patients, the cluster on the caudate nucleus revealed a significant linear relationship with total disease severity scores based on clinical profiles and conventional MRI findings. Higher total disease severity scores were associated with worse disease conditions and correlated with smaller GMVs in the right caudate nucleus (*p* < 0.05)

The relationship between the three WAIS indices (VCI, POI, and WMI) and the significantly smaller GMVs in the patient group compared to the control group revealed significantly positive correlations (*p* < 0.05) (Fig. 2).

Poor VCI, POI, and WMI scores correlated with smaller GMVs in the right thalamus and right superior temporal gyrus. Poor WMI scores correlated with smaller GMVs in the right precuneus and left putamen. Poor VCI and POI scores correlated with smaller GMVs in the right caudate nucleus.

The increased total disease severity scores, based on clinical profiles and conventional MRI findings at the time of admission that indicated more severe disease, correlated with smaller GMVs in the right caudate nucleus (r = -0.752, *p* = 0.003). The higher conventional MRI scores also correlated with smaller GMVs in the right caudate nucleus (r = -0.729, *p* = 0.005), right superior middle gyrus (r = -0.58, *p* = 0.038), and right middle temporal gyrus (r = -0.568, *p* = 0.043).

## Discussion

Up to this point, the relationships among initial disease severity, long-term structural changes, and altered cognitive function in patients with TBM have not been fully studied. The present study corroborates the hypothesis that initial disease severity is correlated to long-term structural abnormalities and cognitive function impairments. In the chronic stage, patients with TBM are morphologically different from healthy subjects, and many of the structural deficits in patients correlate with their declines in cognitive function. Individual disease severity assessed in acute illness is related to long-term structural deficits, especially in the right caudate nucleus. These findings underscore the importance of evaluating initial disease severity to predict long-term disease outcomes.

The structural abnormalities in patients with TBM include smaller GMVs in the right thalamus, right superior temporal gyrus, right precuneus, right middle temporal gyrus, left putamen, right caudate nucleus, and right middle temporal gyrus. These regions may be grouped into two parts - cerebral cortices and deep gray matter nuclei. The results may be explained by considering the pathophysiology of TBM. It develops most often when a caseating meningeal or sub-cortical focus, the Rich focus, discharges its contents into the sub-arachnoid space [26]. Brain damage in TBM is due to the combined effects of increased intra-cranial pressure due to hydrocephalus and ischemic infarction caused by peri-arteritis [27]. The lenticulo-striate arteries, middle cerebral arteries, and thalamo-perforators are the most commonly affected vessels, and thus, the basal ganglia, cerebral cortex, pons, and cerebellum are the areas most involved by ischemia in TBM [28, 29]. In a previous study, diminished caudate volumes in patients with hydrocephalus have been reported in a full-brain voxel-based morphometric analysis [30]. The cerebral cortices are close to the subarachnoid space and have been observed to exhibit higher bacterial concentrations and enlarged perivascular spaces, most commonly in the basal ganglia, specifically in the lenticulo-striate arteries [31]. The findings of the current study corroborate these previous observations by providing a more objective investigation in terms of morphology.

The diagnosis, treatment, and prognostication of TBM still pose a formidable challenge, and early diagnosis is still crucial for successful disease management. Excessive immune activation in the sub-arachnoid space is considered to be associated with poor outcomes in TBM, and a previous study has documented the significant relationship between death from TBM and a relatively low CSF white cell count and low CSF/blood glucose ratio at presentation [32]. Therefore, in the present study, the severity of TBM at the time of admission for each patient was evaluated via clinical profile and conventional MRI findings based on identified significant prognostic factors. As such, a higher total disease severity score during acute illness indicates worse disease condition and is correlated with smaller GMV in the right caudate nucleus. This correlation reflects a relationship between poor prognostic factors and long-term structural deficits in the caudate nucleus.

In comparing the patients with TBM to the healthy controls, we found that the smaller GMVs of the patients in the right thalamus, right superior temporal gyrus, right precuneus, left putamen, and right caudate nucleus were associated with worse cognitive function, including verbal comprehension, perceptual organization, and working memory. The verbal comprehension index is a measure of general verbal skills, such as verbal fluency, ability to understand and use verbal reasoning, and verbal knowledge. A previous study related to chronic aphasia revealed a significant relationship between lesions in the posterior superior temporal gyrus (Wernicke’s area) and impaired verbal comprehension function [33].

The perceptual organization index is a measure of non-verbal and in-the-moment reasoning. Structuring of the sensory scene (perceptual organization) profoundly affects what is perceived. In both visual and auditory functions, many cues have been identified to influence perceptual organization, but very little is known about the neural basis of these functions. The working memory system includes areas performing the processes needed by a multi-functioning memory system that must not only store information but also update and manipulate its contents [34]. In previous research, individuals with thalamic lesions been shown to have a variety of cognitive impairments ranging from memory to attention problems. There are known connections between the thalamus and other structures, such as the basal ganglia [35] and the pre-frontal cortex [36], that have been linked to working memory. To date, the results of the present study represent the most complete documentation in assessing neuro-cognitive function impairments of patients with TBM.

Although the body of this research has the undeniable merit of offering valuable insights into cortical involvement in TBM, this study has some limitations. The first is the small number of participants, which was perhaps due to the low incidence of TBM and the severity of its clinical outcomes, which can include death. Neuro-tuberculosis constitutes approximately 0.7 % of all cases of clinical tuberculosis, but has a high mortality rate of up to 27 % [37]. The present study did not enrol patients with severe sequelae. There is uncertainty in assessing imaging findings in critically ill patients and in those with poor prognosis. Second, there is a wide variety of treatments for TBM, including the use of different antimicrobial agents, corticosteroids, and ventricular shunting, which may cause potential bias in interpreting imaging findings.

## Conclusions

In conclusion, multiple domain cognitive impairment may persist in patients with chronic TBM even after appropriate treatment. Worse initial disease severity may contribute to the vulnerability of brain tissue to damage, with subsequent neuropsychological consequences.

## Abbreviations

TBM: Tuberculous meningitis
DARTEL: Diffeomorphic Anatomical Registration Through Exponentiated Lie algebra
VBM: Voxel-based morphometry
MRI: Magnetic resonance imaging
GMV: Gray matter volume
WMV: White matter volume
WAIS: Wechsler Adult Intelligence Scale
VCI: Verbal comprehension index
POI: perceptual organization index
WMI: Working memory index
